# Experimental Study on the Effect of *Aconite* and *Angelica sinensis* on Myocardial Ischemia Rats with Yang Deficiency and Blood Stasis

**DOI:** 10.1155/2020/7027391

**Published:** 2020-04-26

**Authors:** Yongcang Cao, Xiaodong Liang, Changyi Li, Tao Chen, Zhanling Li, Wanfeng Li, Peipei Liu, Guiyong Li, Ran Ma, Yingxue Tang

**Affiliations:** ^1^Shandong University of Traditional Chinese Medicine, 4655 Daxue Road, Jinan 250355, Shandong, China; ^2^Tai'an Hospital of Traditional Chinese Medicine, 58 Dongyue Road, Tai'an 271000, Shandong, China; ^3^China Resources Shandong Pharmaceutical Co., Ltd., 1088 Meili Road, Jinan 250000, Shandong, China

## Abstract

**Objective:**

To investigate the intervention effect and mechanism of Aconite and *Angelica sinensis* on myocardial ischemia rats with Yang deficiency and blood stasis.

**Methods:**

SPF-class SD rats were randomly divided into low-dose and high-dose groups. Each group was divided into control group, model group, and drug-administered group (FZ, DG, FG; 1 : 0.5, 1 : 1, 1 : 2). A rat model was prepared by intraperitoneal injection of hydrocortisone and isoproterenol plus cold stimulation. Each group was given corresponding decoction or distilled water for 14 days. The behavioral changes of rats in each group were observed. The morphological changes of rats cardiomyocytes were observed by HE staining. The average optical density (MOD value) and percentage of positive cells of Bcl-2, Bax, and Akt were determined by immunohistochemical staining method, and PEIs were calculated. Western blot and RT-PCR were used to determine the expression of PI3K, Caspase-3, Akt protein, and gene expression.

**Results:**

The compatibility of Aconite and *Angelica sinensis* improved the morphology of rat cardiomyocytes, increased the PEI values of Akt and Bcl-2 protein, and decreased the PEI values of Bax protein (*P* < 0.01). The compatibility reduced the expression of Caspase-3 protein of rat myocardium and increased the protein expression of p-Akt, PI3K, and p-PI3K (*P* < 0.01). The compatibility also significantly reduced the expression of Caspase-3 mRNA and increased the expression of PI3K mRNA and Akt mRNA (*P* < 0.05 or *P* < 0.01), and the effect of high-dose FG (1 : 2) group is the best.

**Conclusions:**

The method of preparing a rat model of myocardial ischemia with Yang deficiency and blood stasis was feasible. The compatibility of Aconite and *Angelica sinensis* reduced myocardial fibrosis and inflammatory reaction, protected ischemic cardiomyocytes, and reduced myocardial injury, whose mechanism may be related to the regulation of PI3K/Akt pathway. The compatible group had better intervention effects than Aconite or *Angelica sinensis* alone. The best one was high-dose FG (1 : 2).

## 1. Introduction

Myocardial ischemia (MI) is more common in coronary heart disease (CHD), which refers to heart disease caused by myocardial ischemia or hypoxia caused by coronary atherosclerosis and functional changes; it is a common disease with extremely high mortality, which belongs to the category of “thoracic,” “heartache,” and “syncope” of Chinese medicine. According to the results of modern pharmacological research, Chinese medicine Aconite has the functions of strengthening heart, boosting blood pressure, lowering blood sugar, and regulating nerve disorder and immune system, in addition to its antimyocardial ischemia, antithrombus, antiarrhythmia, antishock, antiaging, antitumor, and analgesic effects [1, 2]. Modern research has confirmed that Aconite can promote myocardial contraction, expand cardiac output, and increase myocardial oxygen consumption. Aconite alkaloids can protect the ischemic myocardium by regulating the expression of related proteins such as energy metabolism, cell repair, and antioxidant emergency [3]. The above effects can be used to treat acute myocardial infarction and heart failure, which is consistent with the efficacy of Aconite in “reviving the Yang for resuscitation.” Chinese medicine *Angelica sinensis* is also a commonly used drug in the cardiovascular system. *Angelica sinensis* extract powder can stabilize mitochondrial membrane potential, downregulate proapoptotic protein BAD, inhibit Cyt c release and Caspase-3/9 activity, and thereby inhibit cardiomyocyte apoptosis; JNK inhibitor (SP600125) can block the inhibition of Caspase-3 activation by *Angelica sinensis* [4, 5]. *Angelica sinensis* ferulic acid can significantly protect myocardial cells from hypoxia and reoxygenation injury by increasing NO production, inhibiting platelet aggregation and proliferation, and improving myocardial cell survival rate; *Angelica sinensis* and its ferulic acid can reduce cerebral infarction area in MCAO model rats by improving neurological deficit score, blood flow, and SOD activity [6, 7].

Jin et al. [8] confirmed that *Angelica sinensis* is highly compatible with other 22 traditional Chinese medicines. Among them, there are more studies on the compatibility of *Angelica sinensis* with Astragalus and less studies on the compatibility of *Angelica sinensis* with Aconite. Based on the literature retrieval analysis of Web of Science, PubMed, ScienceDirect, and other databases, this paper attempts to explore the research value of Aconite and *Angelica sinensis* in the treatment of myocardial ischemia.

## 2. Materials and Methods

### 2.1. Animals and Treatment

Low-dose group (LDG)/high-dose group (HDG) consisted of 70 female/male healthy SPF SD rats, weighing 180–210 g, provided by Jinan Pengyue Experimental Animal Breeding Co. Ltd., Shandong, China, with licensed certificate number SCXK (Lu) 20140007. The laboratory and breeding room are provided by the Basic TCM Laboratory of Shandong University of Traditional Chinese Medicine, China.

According to the “Pharmacological Experimental Methodology” edited by Professor Xu [9], the amount of gavage was calculated by the equivalent dose of body surface area. The LDG group and HDG group were 1.35 g/kg and 2.7 g/kg, respectively.

#### 2.1.1. LDG Group

According to literature method [10] and pre-experiment, after 7 days of adaptive feeding, 8 SD rats were selected as the normal control group by random number table method, which were injected intraperitoneally with the same volume of 0.9% sodium chloride solution; the remaining 62 rats were injected with hydrocortisone sodium succinate 25 mg/kg/d intraperitoneally and, 2 hours later, with isoproterenol hydrochloride 5 mg/kg/d intraperitoneally. After 1 hour, cold stimulation was applied in ice bag environment for 1 h/d for 14 days.

The behavioral changes of rats and the variation amplitude of ST segment (≧0.1 mV) were observed as the successful markers of the model. Forty-nine rats were successfully established, which were divided into model group (9 rats) and administration group (8 rats) by random number table. Rats in control group and model group were given 0.9% sodium chloride solution of the same volume by gavage; rats in each administration group were given corresponding decoction once a day for 14 days. In HDG group, 48 rats were successfully modeled and divided into groups of 8 rats each by random number table. All procedures were approved by the Faculty of Medicine and Health Sciences Ethics Committee for Animal Research of Shandong University of Traditional Chinese Medicine (Ethics no. SDUTCM 2018071501). Every effort was made to minimize pain of the animals.

### 2.2. Drugs

Aconite is a processing product for the seed roots of *Aconitum carmichaelii* Debx., a perennial herb of Ranunculaceae, batch number: 1601232056. *Angelica sinensis* is a root processing product of *Angelica sinensis* (Oliv.) Diele, batch number: 160111. The above Chinese medicines were purchased from Beijing Tongrentang Co., Ltd. (Jingshi Road, Jinan, Shandong, China) and were identified by Professor Li Feng of the biopharmaceutical system of SDUTCM, in line with the 2015 edition of the “Pharmacopoeia of the People's Republic of China” [11]. FG decoction (FG 1 : 0.5, 1 : 1, 1 : 2) was as follows: Aconite was first decocted for 1 hour, with *Angelica sinensis* being added for 30 minutes, second decocted for 1h, and then concentrated after merging filtrate. FZ decoction (FZ) was as follows: first decocting for 1.5 hours, second decocting for 1 hour, and then concentrating after merging filtrate for 1.0 g/ml (calculated by Aconiti). DG decoction (DG) was as follows: first and second decocting for 30 minutes and then concentrating after merging filtrate for 1.0 g/ml (calculated by *Angelica sinensis*). The decoctions were kept in a refrigerator at 2–4°C for reserve.

### 2.3. Behavioral Changes

Behavioral changes including food intake, water intake, mental state, and activity were observed before and after the establishment of models and administration of drugs.

### 2.4. Tissue Collection

After the heart was extracted and rinsed in saline, it was dried with absorbent paper. The left ventricle was cut off, quickly collected, and divided into two parts. One part was rinsed with normal saline, dried, and fixed with 4% paraformaldehyde/0.1 M PBS stationary solution. After dehydration, wax immersion, and paraffin embedding, 5-micron thick tissue sections were prepared for HE staining and immunohistochemical detection. The other part was rinsed with normal saline, dried, stored in a freezer tube, and transferred to the ultralow temperature refrigerator at −80°C for Western blot and RT-PCR detection.

### 2.5. HE Staining

The steps include the following: paraffin section dewaxing by xylene; descending gradient ethanol dewaxing; hematoxylin and eosin staining (Solarbio, Beijing, China); ascending gradient ethanol dehydration; xylene transparency; neutral gum seals; preparing sections and observing them under an optical microscope. Analysis was performed with ZEN 1.01.0 Imaging analysis software (Carl Zeiss Microscopy GmbH, German).

### 2.6. Immunohistochemistry

The steps include the following: paraffin section dewaxing and water entry; 3% methanol hydrogen peroxide incubation at room temperature; high pressure repair; dripping antibody I; dripping antibody II; dripping DAB dyeing solution; hematoxylin counterstaining; microscopic observation; image analysis: the processing system consists of CMOS (Japan, OLYMPUS) and special software (Image-Pro Plus, Media Cybernetics, USA). For each slice (×400), 10 fields of view were randomly selected to determine the MOD value of cell positive staining and the percentage of positive cells, PEI (protein expression index) was calculated: PEI = MOD value × percentage of positive cells.

### 2.7. Western Blot Analysis

According to the test results of related indicators in the early stage, the curative effect of HDG group was superior to that of LDG group. The late Western blot and RT-PCR experiments were performed only in the HDG group.

Western liquid mixtures are 1.5 M Tris-HCl (pH = 8.8), 1 M Tris-HCl (pH = 6.8), 10% (w/v) SDS, acrylamide storage solution, 4x separating gel buffer, 4x concentrated gel buffer, 10% ammonium persulfate, electrophoretic buffer, 5x sample buffer, Coomassie brilliant blue decolorizing solution, Coomassie brilliant blue dyeing solution, 0.01 M phosphate buffer (PBS), electroconversion fluid, and sealing fluid.

The main steps include protein extraction, quantification, preparation of SDS-PAGE gel, denaturation and electrophoresis of the sample, electrophoretic transfer and detection, selection of appropriate exposure time, and imaging by the chemiluminescence imaging system (UVP gel imaging system, GDS-8000 System, Thermo Fisher Technology Co. Ltd., China)

### 2.8. RT-PCR

Primer design was as follows [12, 13]: PI3K: ACAAAGCCGAGAACCTAT/GACTTCGCCATCTACCAC; Akt: TCACCTCTGAGACCGACA/AGGAACTGGGAAAGT;Caspase-3: GCGGTATTGAGAGACAGACACA/AAGCATAGGAAGTCGG; beta-actin: CCTAGACTTCGAGCAAGAGA/GGAAGGAAGGCTGGAAGA. Total RNA was extracted by Trizol method, RNA electrophoresis, and reverse transcription; determined by fluorescence quantitative PCR (SLAN8.0, Hongshi Medical Technology Co. Ltd, Shanghai, China); and quantitatively analyzed by 2^△△CT^ method. The following antibodies can be used: rabbit anti-rat Bax (ZSGB-BIO, cat no. sc-74800), rabbit anti-rat Akt1/2 (ZSGB-BIO, cat no. sc-16190), antibody beta-actin (ZSGB-BIO, cat no. TA-09), rabbit anti-rat Caspase-3 (Proteintech, cat no. 19677-1-AP), rabbit anti-rat Bcl-2 (Protech, cat no. 12789-AP), rabbit anti-rat PI3K (Bioss, cat no. bs-0128R), rabbit anti-rat p-PI3K (Bioss, cat no. bs-6417R), and rabbit anti-rat p-Akt (Thr308) (Bioss, cat no. bs-2720R).

### 2.9. Statistical Analysis

All data were analyzed by SPSS 22.0 professional software (SPSS, USA) and expressed as mean ± standard deviation ($\bar{x}\pm s$). One-way ANOVA was performed on each group of data, and the homogeneity test of variance was performed. *P* < 0.05 or *P* < 0.01 indicates that the difference is statistically significant.

## 3. Results

### 3.1. Behavioral Changes

Rats in the control group had normal feeding, drinking, and activities and were responsive and mentally active. In the model group, the rats had less food intake, more drinking water, more urine, less activity, lack of energy, aggravation and contraction, uneven breathing, and slow weight gain. Rats in each drug-administered group increased their intake and drinking water, improved mental state, with gentle breathing, and accelerated weight gain, especially in the FG (1 : 1) and FG (1 : 2) rats of the HDG group.

### 3.2. HE Staining

In the control group, the distribution of myocardial cells was regular and morphologically intact; the myocardial fibers were arranged neatly, the structure was clear, and the nucleus was oval in the center of the cell. Compared with the control group, the myocardial structure of the model group was severely damaged and there were different degrees of necrosis, including focal or flaky necrosis. Compared with model group, the three groups of FZ, DG, and FG (1 : 0.5) improved in varying degrees. There were fibrotic areas, disordered arrangement of myocardial fibers, reduced flake necrosis, inflammatory cell infiltration, and little dense nuclei. The two groups of FG (1 : 1) and FG (1 : 2) improved significantly, and FG (1 : 2) group had the best effect.

### 3.3. PEI of Akt, Bcl-2, and Bax

**Figure 1:**
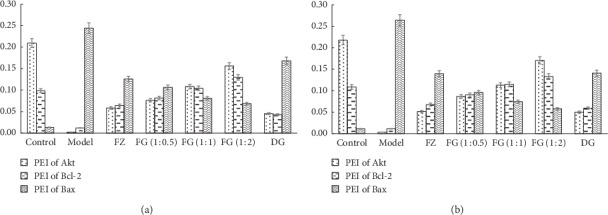
PEI values of each group (Akt/Bcl-2/Bax). (a) LDG. (b) HDG.

As shown in Figure 1, compared with the control group, the PEI values of the Akt and Bcl-2 proteins in the model group were significantly decreased, and the PEI values of the Bax protein were significantly increased (*P* < 0.01). Compared with the model group, the PEI values of Akt and Bcl-2 proteins in the rats in each administration group increased, and the PEI values of Bax protein decreased (*P* < 0.01). Compared with the FZ and DG groups, the FG (1 : 0.5), FG (1 : 1), and FG (1 : 2) groups of Aconite and *Angelica sinensis* were more significantly improved (*P* < 0.05 or *P* < 0.01).

### 3.4. Protein Expression of Caspase-3, PI3K, p-Akt, and p-PI3K

**Figure 2:**
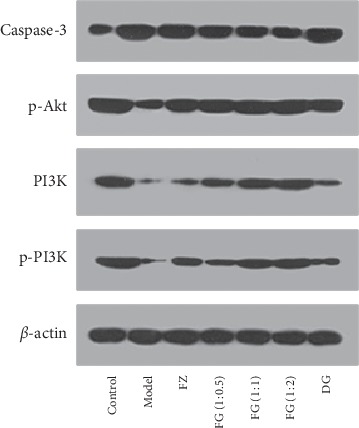
The expression of myocardial protein in rats of each group.

**Figure 3:**
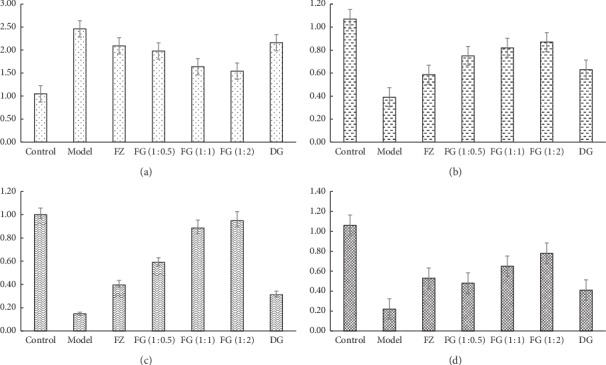
Expression of caspase-3/p-Akt/PI3K/p-PI3K in myocardium of rats in each group. (a) Relative level of caspase-3 protein. (b) Relative level of p-Akt protein. (c) Relative level of PI3K protein. (d) Relative level of p-PI3K protein.

As shown in Figure 2, Table 1, and Figure 3, compared with the control group, the expression of Caspase-3 protein in model group was significantly higher, while the expression of PI3K, p-Akt, and p-PI3K protein was significantly lower (*P* < 0.01). Compared with model group, the expression of Caspase-3 protein in myocardium was significantly decreased in each administration group (*P* < 0.01), and the expression of PI3K, p-Akt, and p-PI3K protein was significantly increased (*P* < 0.01). Compared with FZ and DG groups, the expression of Caspase-3 protein in myocardium of FG (1 : 0.5), FG (1 : 1), and FG (1 : 2) groups was significantly improved (*P* < 0.05 or *P* < 0.01). FG (1 : 2) group was better than FG (1 : 0.5) and FG (1 : 1).

### 3.5. Gene Expression of Caspase-3, PI3K, and Akt

**Figure 4:**
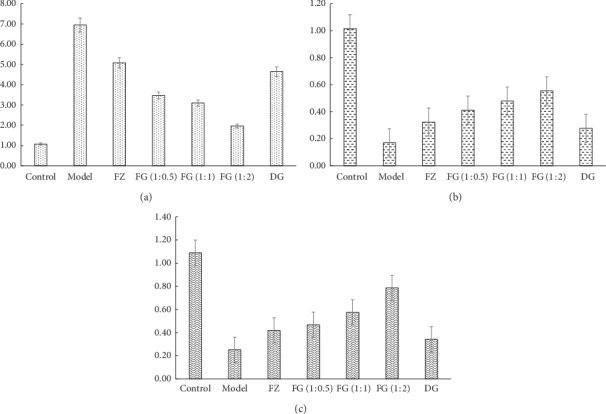
The expression level of caspase-3/PI3K/Akt mRNA of rats in each group. (a) Relative level of caspase-3 mRNA. (b) Relative level of PI3K mRNA. (c) Relative level of Akt mRNA.

As shown in Table 2 and Figure 4, compared with the control group, the expression of Caspase-3 mRNA in myocardium of rats in model group increased significantly, and the expression levels of PI3K mRNA and Akt mRNA decreased significantly (*P* < 0.01). Compared with model group, the expression of Caspase-3 mRNA in myocardium of rats in each administration group decreased, while the expression of PI3K mRNA and Akt mRNA increased (*P* < 0.01). Compared with FZ and DG groups, the expression of Caspase-3 mRNA in myocardium of rats in FG (1 : 0.5), FG (1 : 1), and FG (1 : 2) groups decreased; PI3K mRNA expression significantly increased (*P* < 0.01); and Akt expression significantly increased (*P* < 0.01, except FG (1 : 0.5)). Comparing the compatibility groups of Aconite and *Angelica sinensis*, we found that the expression levels of Caspase-3 mRNA and Akt mRNA in FG (1 : 2) group were better than those in FG (1 : 1) and FG (1 : 0.5), while the expression of PI3K mRNA was not significantly different (*P* < 0.05).

## 4. Discussion

In recent years, the incidence of myocardial ischemia has gradually increased, and the treatment methods have been studied intensively. Among them, traditional Chinese medicine has played a unique role and has attracted more and more researchers' attention [14]. ISO is often used to prepare myocardial ischemia model. High-dose or long time injection of ISO can overexcite cardiac beta-1 adrenergic receptor and increase myocardial contractility, cardiac output, and myocardial oxygen consumption, leading to myocardial ischemia and hypoxia. ISO also can activate cardiac beta 2 receptor and excite vascular alpha receptor, dilate peripheral blood vessel, reduce cardiac return blood volume, and aggravate myocardial ischemia and hypoxia, whose mechanism may be related to oxidative stress, disorder of mitochondrial energy metabolism, and regulation of proapoptotic gene and antiapoptotic gene [15–18]. The minimum dose of ISO is usually 0.5 mg/kg/d and the maximum dose is 500 mg/kg/d. The dose of 3–5 mg/kg/d is often used to prepare myocardial ischemia model [19–22]. With hydrocortisone and cold stimulation, which are commonly used to prepare Yang deficiency and blood stasis, the myocardial ischemia model of Yang deficiency and blood stasis was studied in this paper.

In the middle of the 20th century, Glucksmann, Kerr, Lockshin, and other scientists proposed the concept of cell death, apoptosis, and programmed cell death [23]. Phosphatidyl-inositol-3-kinase/protein kinase B (PI3K/Akt) pathway is an important signal transduction pathway involved in cell apoptosis [24]. According to the structure of subunits and substrates, PI3K can be divided into types I, II, and III [25], having the activity of lipid kinase and protein kinase. The most widely studied type is type I. When activated, PI3K promotes the production of second messengers of lipid products 3,4-diphosphate/3,5-diphosphate/3,4,5-triphosphate phosphatidyl-inositol [PI(3,4/3,5/3,4,5)P3]; Akt's AH/PH domain has a high affinity for PIP3 and is activated after binding. After phosphorylation of upstream protein kinases (e.g., PDK1), Akt translocation occurs subsequently, resulting in catalytic activity and exposure of binding sites. Akt can exert antiapoptotic effects by regulating eNOS, Caspase-3/9 protein kinase, GSK-3 beta and BAD protein [26–29]. Akt is the central link of this pathway, being not only the direct target of the downstream, but also the most important target protein, which has protective effects on myocardial tissue damage induced by various factors [30]. Caspase-3 is the key executor and effector in the process of apoptosis, plays an important role in inducing apoptosis of cardiomyocytes, and has gradually become a potential target for antiapoptotic therapy [31]. The process of cardiomyocyte apoptosis involves very complex molecular biological mechanisms. By regulating the related signaling pathways, cardiomyocyte damage and cardiomyocyte apoptosis can be reduced [32].

TCM (traditional Chinese medicine) treatment of cardiovascular diseases can protect myocardial ischemia by improving the structure and function of the heart, improving coronary blood flow, reducing myocardial oxygen consumption and antioxidative stress response, improving microcirculation, preventing ventricular remodeling, and regulating the expression of proteins and genes in myocardial tissue [33, 34]. Modern pharmacological studies have confirmed that Aconite has many functions, such as cardiotonic, antimyocardial ischemia, antithrombosis, antiarrhythmia, and antishock functions. Aconite can also improve the expression of apoptosis-related factors Bax, Bcl-2, Cyt c, and Caspase-3/-9 [35, 36]. The diester alkaloids of Aconite have obvious cardiac effects. Noraconitine, aconitine, coumarin, uracil, and norsaline can alleviate myocardial injury and protect myocardial cells. The water/ethanol/n-butanol extract of Aconite can specifically prevent the occurrence of ventricular fibrillation induced by chloroform. Aconite-containing decoction can reduce myocardial infarct size, suppress left ventricular remodeling, and improve cardiac function [37]. *Angelica sinensis* can inhibit Ang II-induced changes in mitochondrial membrane potential and Cyt c release in H9c2 cells and inhibit Caspase-3/9 activity, whose mechanism may be related to JNK and PI3K [2, 38].

HE staining showed that the morphology and structure of myocardial tissue in the model group significantly changed, which indicates that the myocardial ischemia modeling method used in this article is successful. Aconite compatibility with *Angelica sinensis* improved the pathological changes of rat cardiomyocytes and reduced myocardial fibrosis and inflammatory response. The compatibility group is better than the single-use group (FZ group and DG group), especially the high-dose FG (1 : 2) group.

Immunohistochemistry showed that values of Bcl-2 and Akt PEI of myocardial tissue of model group significantly decreased, while values of Bax PEI significantly increased (*P* < 0.01), showing significant myocardial damage, which also indicates that the myocardial ischemia modeling method used in this article is successful. Aconite compatibility with *Angelica sinensis* reversed the morphological changes of Bcl-2, Akt, and Bax caused by modeling. The compatibility group was better than the single-use group, especially the high-dose FG (1 : 2) group. Western bolt and RT-PCR experiments showed that expressions of Caspase-3 protein and Caspase-3 gene increased; expressions of p-Akt, PI3K, p-PI3K protein, PI3K, and Akt gene significantly decreased in model group (*P* < 0.01). Aconite compatibility with *Angelica sinensis* reversed the changes in Caspase-3, p-Akt, PI3K, p-PI3K, and Akt protein expression and gene expression caused by modeling. The compatibility group was better than the single-use group, especially high-dose FG (1 : 2) group.

## 5. Conclusions

We concluded that it is feasible to prepare a rat model of myocardial ischemia with Yang deficiency and blood stasis by hydrocortisone sodium succinate and isoprenaline hydrochloride plus cold stimulation. Aconite-compatible *Angelica sinensis* exerted a good antimyocardial ischemia effect through the PI3K/Akt signaling pathway. The compatible group has better intervention effects than Aconite or *Angelica sinensis* alone. The best one was high-dose FG (1 : 2).

## Figures and Tables

**Table 1:** The expression of myocardial protein in rats of each group.

Groups	*n*	Caspase-3	p-Akt	PI3K	p-PI3K
Control	6	1.05 ± 0.06	1.07 ± 0.06	1.10 ± 0.11	1.06 ± 0.11
Model	6	2.46 ± 0.14	0.39 ± 0.05	0.17 ± 0.03	0.22 ± 0.03
FZ	6	2.09 ± 0.12	0.59 ± 0.05	0.39 ± 0.03	0.53 ± 0.08
FG (1 : 0.5)	6	1.98 ± 0.10	0.75 ± 0.03	0.58 ± 0.09	0.48 ± 0.06
FG (1 : 1)	6	1.64 ± 0.21	0.82 ± 0.04	0.88 ± 0.03	0.65 ± 0.07
FG (1 : 2)	6	1.54 ± 0.13	0.87 ± 0.04	0.92 ± 0.03	0.78 ± 0.05
DG	6	2.16 ± 0.19	0.63 ± 0.03	0.30 ± 0.06	0.41 ± 0.03

**Table 2:** Expression levels of caspase-3 mRNA, Akt mRNA, and PI3K mRNA in each group.

Groups	*n*	Caspase-3	PI3K	Akt
Control	6	1.07 ± 0.12	1.08 ± 0.11	1.09 ± 0.12
Model	6	6.95 ± 0.84	0.17 ± 0.01	0.25 ± 0.05
FZ	6	5.08 ± 0.86	0.32 ± 0.06	0.42 ± 0.10
FG (1 : 0.5)	6	3.47 ± 0.86	0.41 ± 0.08	0.47 ± 0.08
FG (1 : 1)	6	3.09 ± 0.64	0.48 ± 0.08	0.58 ± 0.06
FG (1 : 2)	6	1.98 ± 0.32	0.55 ± 0.06	0.79 ± 0.09
DG	6	4.65 ± 0.62	0.28 ± 0.03	0.34 ± 0.08

## Data Availability

The datasets used to support the findings of this study are available from the corresponding author upon request.
